# The incidence of tuberculous pleurisy in mainland China from 2005 to 2018

**DOI:** 10.3389/fpubh.2023.1180818

**Published:** 2023-06-15

**Authors:** Shuhan Chen, Yi Wang, Yuan Zhan, Changyu Liu, Qi Wang, Jie Feng, Yufeng Li, Huilong Chen, Zhilin Zeng

**Affiliations:** ^1^Second Clinical College, Tongji Hospital, Tongji Medical College, Huazhong University of Science and Technology, Wuhan, China; ^2^Reproductive Medicine Center, Tongji Hospital, Tongji Medical College, Huazhong University of Science and Technology, Wuhan, China; ^3^Department of Respiratory and Critical Care Medicine, Tongji Hospital, Tongji Medical College, Huazhong University of Science and Technology, Wuhan, China; ^4^Department of Thoracic Surgery, Tongji Hospital, Tongji Medical College, Huazhong University of Science and Technology, Wuhan, China; ^5^Department of Geriatrics, Tongji Hospital, Tongji Medical College, Huazhong University of Science and Technology, Wuhan, China; ^6^Department of Social Medicine and Health Management, School of Public Health, Huazhong University of Science and Technology, Wuhan, Hubei, China; ^7^Department and Institute of Infectious Diseases, Tongji Hospital, Tongji Medical College, Huazhong University of Science and Technology, Wuhan, China

**Keywords:** tuberculous pleurisy, incidence, spatiotemporal distribution, the annual percent change, China

## Abstract

**Background:**

Currently, tuberculous pleurisy (TP) remains a serious problem affecting global public health, including in China. Our purpose was to comprehensively understand and identify the incidence of TP in mainland China between 2005 and 2018.

**Methods:**

The data on registered TP cases from 2005 to 2018 were acquired from the National Tuberculosis Information Management System. We analyzed the demographics, epidemiology, and time-space distribution of TP patients. Then, the effects of potentially influential factors on TP incidences, such as medical expenses per capita, GDP per capita, and population density, were assessed using the Spearman correlation coefficient.

**Results:**

The incidence of TP increased in mainland China from 2005 to 2018, with a mean incidence of 2.5 per 100,000 population. Interestingly, spring was the peak season for TP, with more notified cases. Tibet, Beijing, Xinjiang, and Inner Mongolia had the highest mean annual incidence. A moderate positive relationship was found between TP incidence, medical expenses per capita, and GDP per capita.

**Conclusions:**

The notified incidence of TP had an elevated trend from 2005 to 2018 in mainland China. The findings of this study provide insight into the knowledge of TP epidemiology in the country, which can help optimize resource allocation to reduce the TP burden.

## Introduction

Tuberculosis (TB) is an ancient communicable disease and remains one of the most severe public health issues worldwide, with ~10.1 million TB cases in 2020 (1). Mycobacterium TB, in addition to pulmonary damage, can lead to extrapulmonary TB (EPTB), including many subtypes, namely pleural, peripheral lymph node, and osteoarticular (2). Compared with pulmonary TB (PTB), EPTB has received less attention from public health entities (3). Tuberculous pleurisy (TP) is reported to be the most common subtype and is highly prevalent in many developing countries such as Romania, Pakistan, and China (4–6). Its clinical manifestations are diverse, and most TP patients present with subacute onset of fever, cough, pleuritic chest pain, and breathlessness (7). In China, TP accounts for 49.97% of all EPTB patients and causes numerous socioeconomic burdens (6). To date, there has been no in-depth national epidemiological study on TP in China.

Bacillus serves as an indicator of the inverse progress of the human race, specifically concerning TB. Tuberculous is one of the most prevalent infectious diseases, principally affecting the world's poorer countries (8). In resource-poor countries such as Lima, Peru, or Senegal, the challenges in diagnosis and treatment have aggravated the epidemiology dilemma and disease burden relating to TB (9–11). In contrast, TB cases are low in high-income regions (12, 13). The significant correlation between TB infection and socioeconomic status is quite apparent. Furthermore, Dye et al. identified multiple determinants of TB incidence rate, including human development index, child mortality, and access to improved sanitation (14). Thus far, very few researchers have reported the associations between TP incidence and the factors mentioned above.

China has the third-highest number of TB cases globally (1). Therefore, in this study, we aimed to examine the epidemiology of TP at the national and provincial levels in mainland China. The potentially influential factors on TP incidence, such as health expenditure, per capita GDP, and population density, were investigated.

## Materials and methods

### Case definition

Tuberculous pleurisy refers to any bacteriologically confirmed or clinically diagnosed case of TB involving pleura other than the lungs. Bacteriological confirmation is based on the positive result of biological specimens through hydrothorax smear microscopy, culture, or WHO-approved rapid diagnostics. Clinical diagnosis is based on imaging (such as X-ray abnormalities suggestive of pleural effusion), clinical manifestations (fever, cough, or chest tightness), suggestive histology, and extrapulmonary cases without laboratory confirmation (15, 16).

To better understand the age distribution of TP, we divided patients into five age groups: 0–14, 15–29, 30–44, 45–59, and 60 years and above. We then explored the variation in trend in the number of cases and incidence by age and sex over time. The 31 provinces in mainland China were categorized into three regions (western, central, and eastern). The seasons were designated as spring (March, April, May), summer (June, July, August), autumn (September, October, November), and winter (December, January, February) (17).

### Data acquisition

In 2005, the Ministry of Health in China had an electronic Tuberculosis Information Management System (TBIMS) that gathered pivotal information on TB cases reported in TB healthcare centers nationwide. The TBIMS data were publicly available at the Data-center of China Public Health Science (18). In total, 472,920 TP patients were documented in TBIMS from 2005 to 2018. The case incidences and demographic information (gender, age, occupation, and residence province) of patients diagnosed with TP were extracted from the system directly. Furthermore, the data on yearly population statistics, medical expenses per capita, and GDP per capita in various provinces were accessed from the National Bureau of Statistics of the People's Republic of China.

### Statistical analysis

The incidence rate was calculated by dividing the number of persons developing a disease by the total time at risk for all people to get the disease (19). We utilized Joinpoint regression models of annual incidence over time to recognize variation points in the temporal tendency of TP (20). The final model applied two-line segments (one joinpoint). The annual percent change (APC) was calculated to determine the direction and amplitude of the trends (20). A series of subsequent analyses were layered based on the periods defined by the change points.

Spearman's correlation coefficients were used to analyze the relationships between TP incidence and health expenditure, per capita GDP, and population density. The maps were made using ECharts software. All statistical tests are two-sided, and the *p*-value < 0.05 was considered statistical significance.

## Results

### Demographic characteristic

Approximately 472,920 patients were infected with TP between 2005 and 2018. The demographic data for these patients are presented in Table 1. The vast majority of cases were men (*n* = 312,185,66.0%) compared with women (*n* = 160,735,34.0%), and the male-to-female ratio was 1.9. Among the five age groups, the proportion of TP patients in the age group of 15–29 years was the highest, followed by the age group of 30–44 years. Besides, 39.2% of TP cases were registered in eastern China. Farmers accounted for 50.1% of all cases.

**Table 1:** The characteristics of patients with tuberculous pleurisy in China from 2005 to 2018.

**Characteristic**	**Patient group**
**Sex**	
Male	312,185 (66.0)
Female	160,735 (34.0)
**Age group, y**	
0–14	9,602 (2.0)
15–29	168,429 (35.6)
30–44	104,338 (22.1)
45–59	89,150 (18.9)
≥60	101,401 (21.4)
**Region**	
Eastern	185,163 (39.2)
Central	156,792 (33.2)
Western	130,965 (27.7)
**Occupation**	
Nursery children	353 (0.1)
Stay-home children	1,298 (0.3)
Students	42,046 (9.8)
Teachers	3,740 (0.9)
Farmers staff	214,106 (50.1)
Commercial service stratum	10,434 (2.4)
Medical staff	3,198 (0.7)
Workers	37,942 (8.9)
Government employees	35,797 (8.4)
Unemployed	48,325 (11.3)
Other	2,9972 (7.0)

TP, tuberculous pleurisy. Data are expressed as the number (percentage) of patients. Percentages have been rounded. The statistical data on occupation was provided from 2005 to 2017.

### Incidence and epidemic pattern

**Figure 1:**
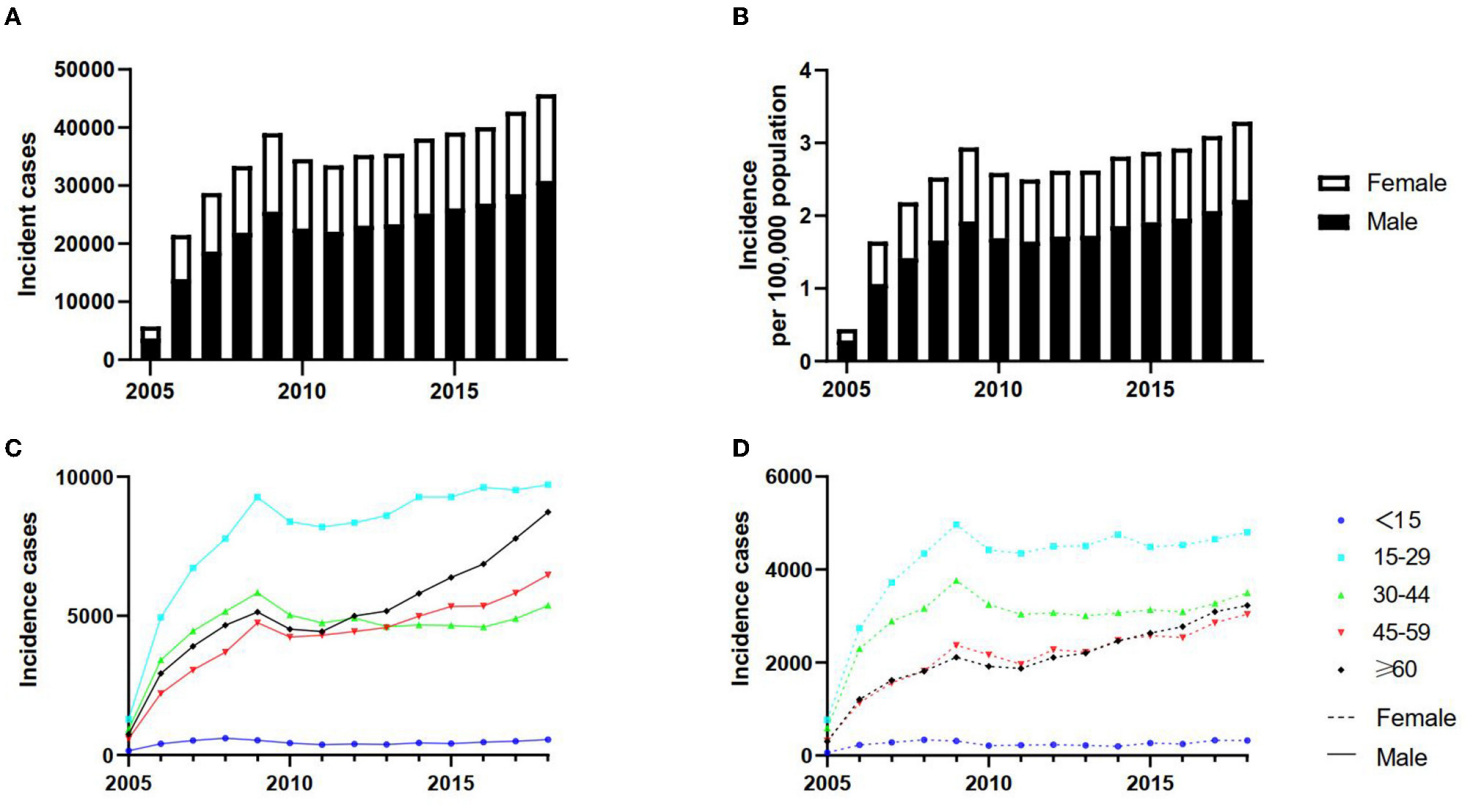
The trends of tuberculous pleurisy incident cases in mainland China from 2005 to 2018. The change in trends of incident cases **(A)**; The change in trends of incidence per 100,000 population **(B)**; the change in trends in different age groups among men **(C)**; the change in trends in different age groups among women **(D)**.

Overall, TP cases increased from 5,725 in 2005 to 45,711 in 2018, with an annual increase of 0.4 per 100,000 population in 2005 to 3.3 per 100,000 population in 2018 (Figures 1A, B). The 14-year mean yearly incidence was 2.5 per 100,000 population. Based on the data, the mean annual incidence doubled from 1.4 per 100,000 population between 2005 and 2007 to 2.8 per 100,000 population between 2008 and 2018 (Supplementary Table 1).

Figures 1C, D reveal that the number of cases was similar across different age groups and genders from 2005 to 2018, consistent with the annual incidence change. While those aged 15–29 accounted for the largest share of TP, those under 15 registered the lowest all the time, regardless of sex. Incident cases of TP increased significantly among men 60 years and older, ranking that age group as the second highest group affected by TP in recent years. Interestingly, during the study period (2005–2018), women aged 30–44 years had the second highest number of TP among different age groups of women. The two curves consisting of 45–59 and ≥60 age groups nearly overlapped in women, with the incidence in both age groups altering roughly the same over time.

**Figure 2:**
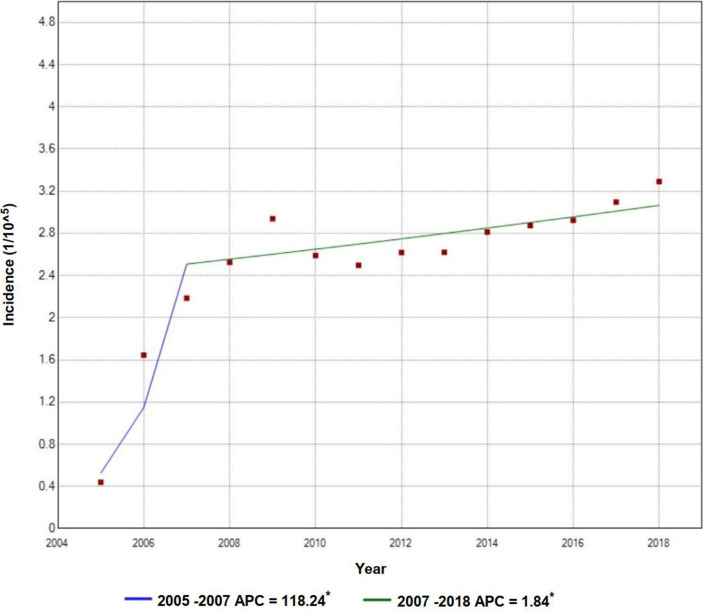
Trends and joinpoints of tuberculous pleurisy incidence. *Indicates that Annual Percent Change (APC) significantly differs from zero at the a = 0.05 level. For the joinpoint model, TP incidence = β0 + β1 (year) + β2 (year + 2007) + E.

**Figure 3:**
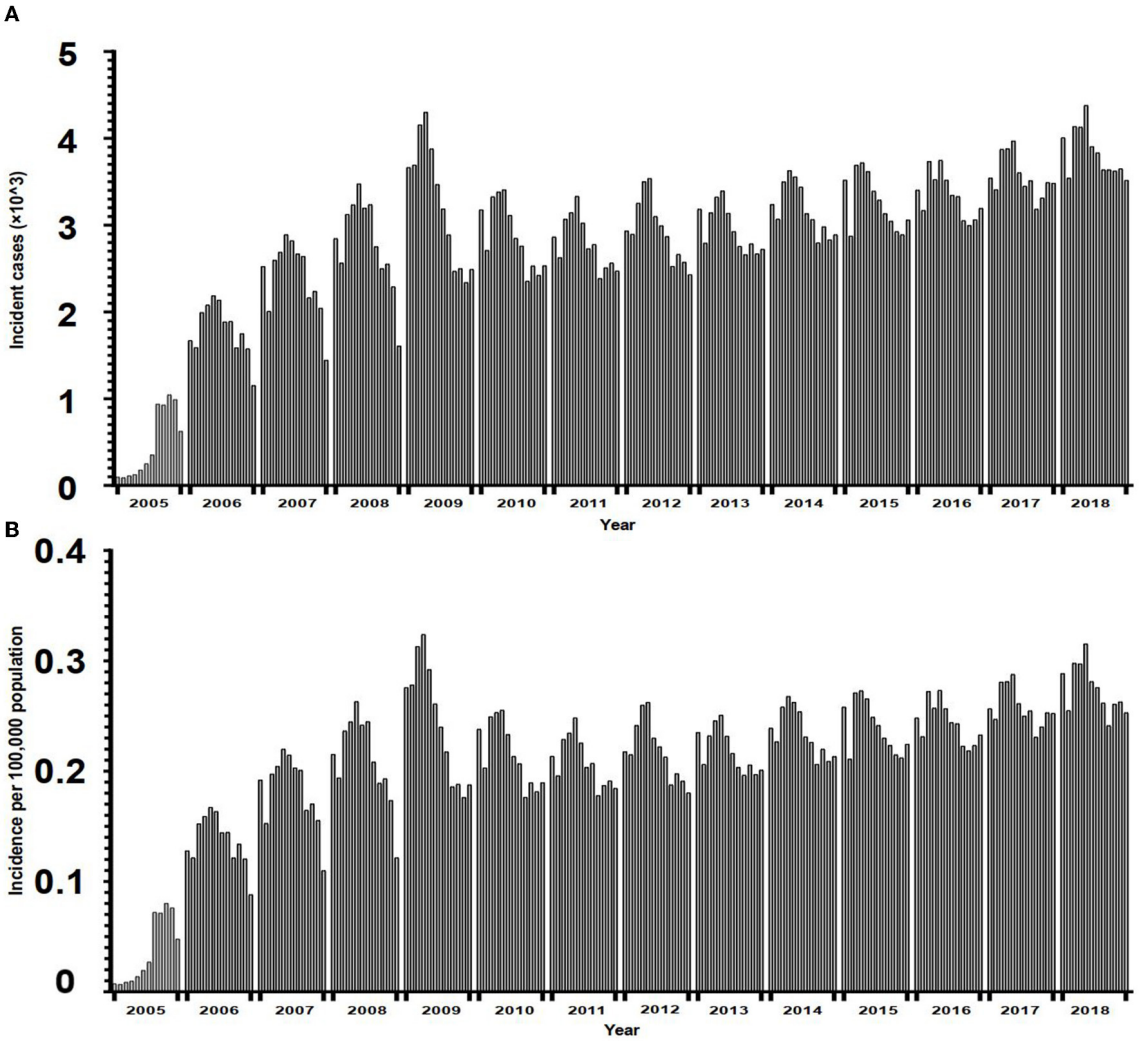
The seasonal distribution of monthly tuberculous pleurisy in China from 2005 to 2018. Incident cases **(A)**, incidence per 100,000 population **(B)**.

The annual incidence of TP rose rapidly from 0.4 per 100,000 population in 2005 to 2.2 per 100,000 population in 2007, with an APC value of 118.2. It then climbed steadily to 3.3 per 100,000 population in 2018 with an APC value of 1.8 (Figure 2). The incidence of TP had obvious seasonality, with notifications being higher in spring, especially in May. Figure 3 shows the sharp spike after January until it reaches the peak in April or May and then drops gradually in the following months days (Figures 3A, B).

### Spatiotemporal distribution

**Figure 4:**
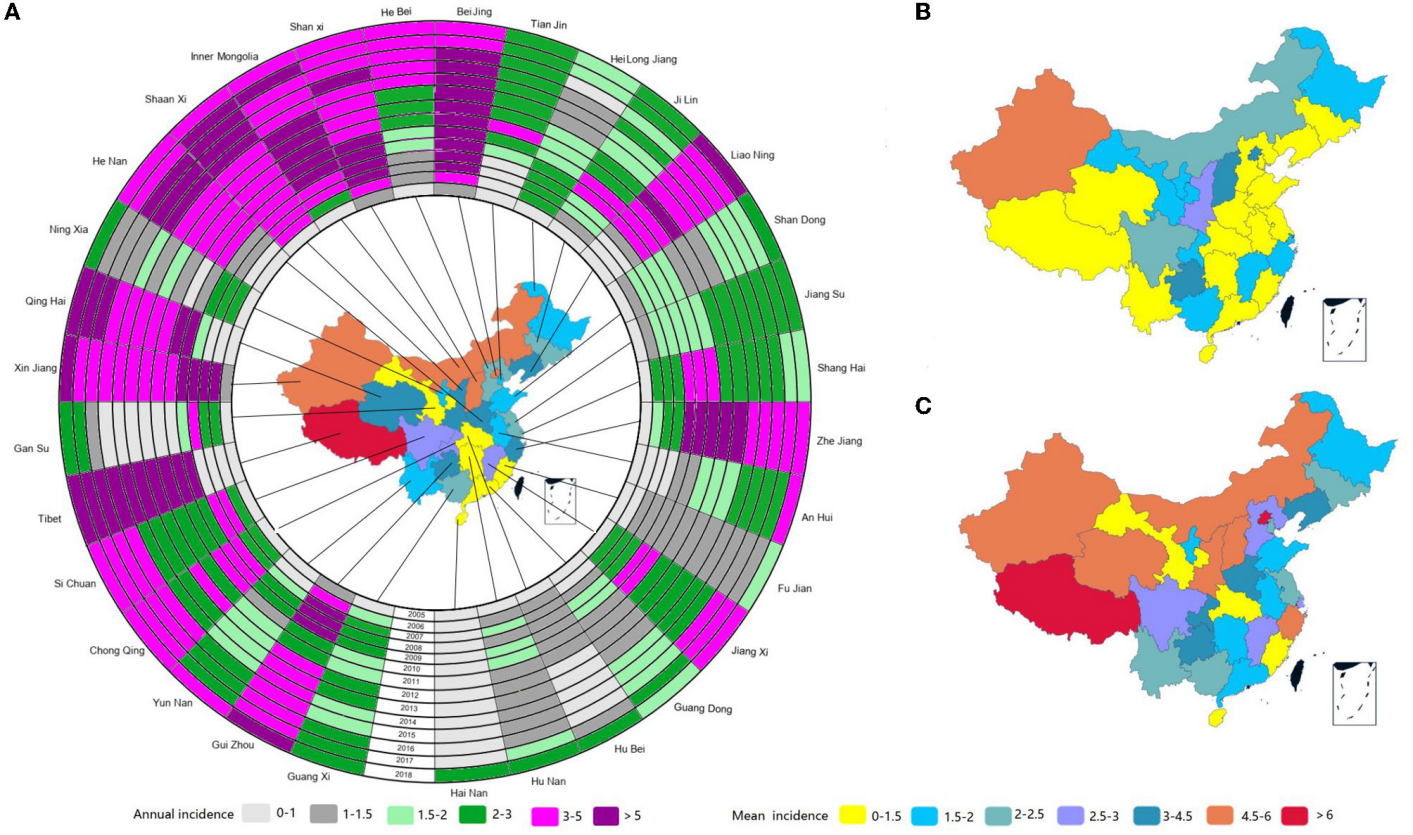
Spatiotemporal distribution of tuberculous pleurisy in China from 2005 to 2018. Map of mean annual incidence per 100,000 population by province from 2005 to 2018 **(A)**; Map of mean annual incidence from 2005 to 2007 **(B)**; Map of mean annual incidence from 2008 to 2018 **(C)**.

The top five provinces in terms of average TP annual incidence between 2005 and 2018 were Tibet (11.4 per 100,000 population), Beijing (5.6 per 100,000 population), Xinjiang (5.1 per 100,000 population), Inner Mongolia (4.9 per 100,000 population), and Shanxi (4.5 per 100,000 population). Comparatively, Hainan (0.6 per 100,000 population), Hubei (1.1 per 100,000 population), Fujian (1.1 per 100,000 population), Guangdong (1.4 per 100,000 population), and Hunan (1.5 per 100,000 population) had low mean annual incidence during the same period (Figure 4A; Supplementary Table 2).

The annual incidence in Tibet and Beijing exceeded 5.0 per 100,000 population for most of the time (nine out of 14 years) (Figure 4A). Most provinces registered an increase in their average yearly incidence (2005–2007 vs. 2008–2018), including Tibet, Beijing, Qinghai, Inner Mongolia, Zhejiang, Yunnan, Tianjin, Hunan, Henan, Hebei, Shanxi, Shaanxi, Liaoning, Jilin, and so on. The most significant jump occurred in Tibet from <1.5 per 100,000 population to >6.0 per 100,000 population, followed by Qinghai from <1.5 per 100,000 population to >4.5 per 100,000 population. In contrast, Ningxia, Xinjiang, Heilongjiang, Gansu, Hainan, Hubei, and Fujian had an unchanged mean annual incidence during the period under study. Xinjiang had a consistently high mean annual incidence (more than 5.0 per 100,000 population) throughout the study period (2005 to 2018), and Hainan, Hubei, and Fujian maintained a relatively consistent low yearly incidence during the same period (Figures 4B, C).

### Potential influencing factors

**Figure 5:**
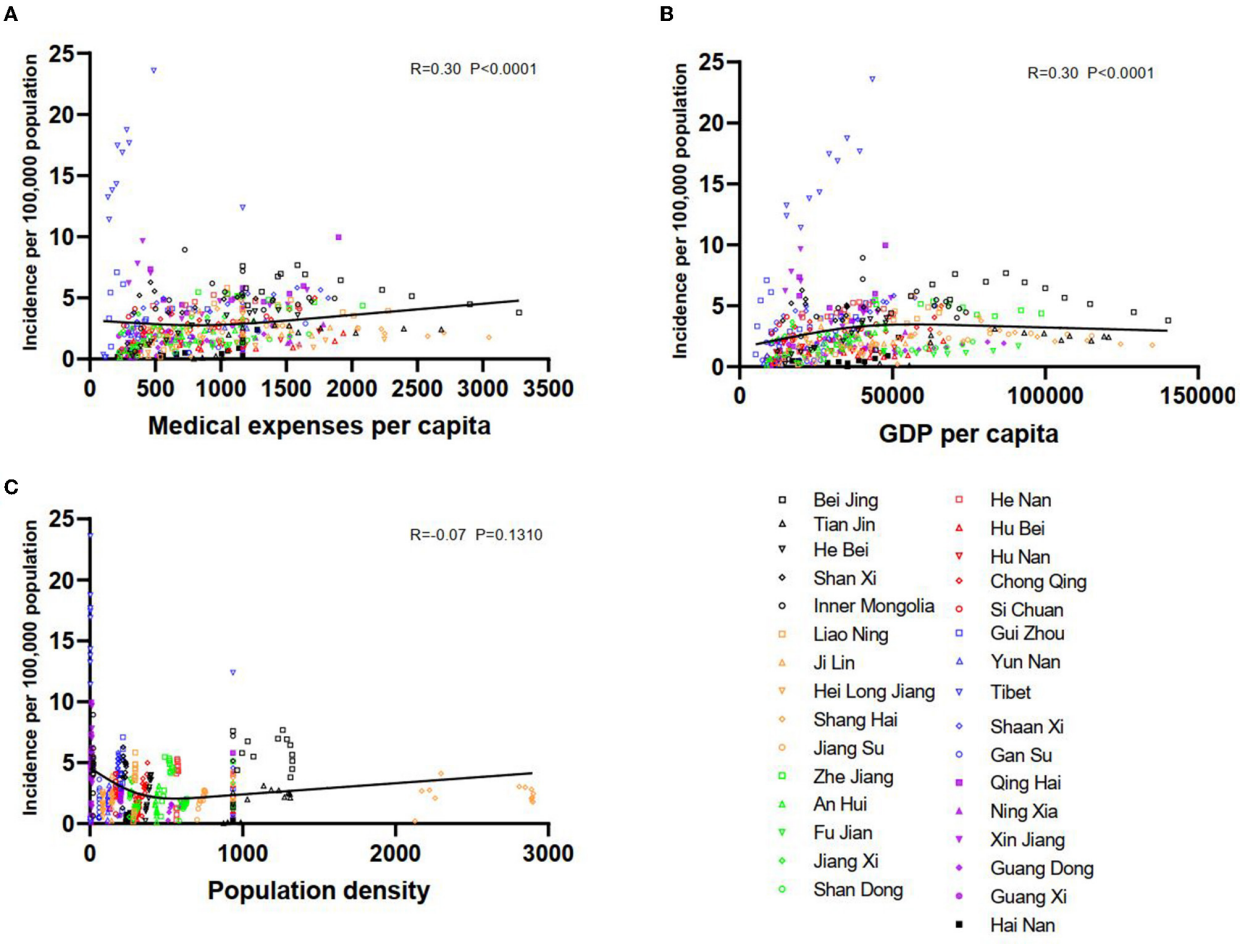
The potentially influential factors of tuberculous pleurisy incidence. The association between the incidence of tuberculous pleurisy and medical expenses per capita **(A)**; The association between the incidence of tuberculous pleurisy and GDP per capita **(B)**; The association between the incidence of tuberculous pleurisy and population density **(C)**.

At the provincial level, factors that may influence TP incidence were examined. We found that medical expenses per capita were positively correlated with the incidence per 100,000 population (R = 0.3, *P* < 0.0001) (Figure 5A). Similarly, GDP per capita was also positively related to incidence per 100,000 population (R = 0.3, *P* < 0.0001) (Figure 5B). However, population density was not proven to correlate with the incidence per 100,000 population (R = −0.07, *P* = 0.1310) (Figure 5C).

## Discussion

In this study, we presented that the incidence of TP increased over time from 0.4 per 100,000 population in 2005 to 3.3 per 100,000 population in 2018. Most cases were concentrated in males, the younger population aged 15–29, and farmers. The incidence of TP in spring was higher than in other seasons. Moreover, Tibet, Beijing, Xinjiang, and Inner Mongolia had higher mean annual incidence and were China's high TP burden provinces. The TP incidence positively correlated with per capita medical expenses and GDP. To our knowledge, our study is the first report on the epidemiological pattern and change trends of TP in mainland China from 2005 to 2018. Our findings provide an insight into TP epidemiology in mainland China, which is of great value to optimizing resource allocation to reduce TP burden.

We observed a disparity between male and female patients; women had lower rates of TP. Our results were in line with previous studies, demonstrating that TP was more common in men (5, 16, 21). Possible explanations for these gender differences included the following factors: smoking, alcohol and drug abuse, sex hormones, and genetic background (22, 23). Moreover, TB cases among men were reported to surpass those found in women by a ratio of 2 to 1 in most countries (22, 24). However, in the literature, EPTB was more commonly reported in female patients (25). As a common manifestation of extrapulmonary mycobacterium TB infection, the sex distribution of TP was different from that of EPTB, one potential reason being that TP occurred secondary to PTB (26); therefore, the analogous gender preference between TP incidence and PTB incidence.

An earlier study undertaken in Turkey showed that young adults were more likely to be affected by PTB or EPTB (27). Similarly, our data suggested that the proportion of TP in the population aged 15–29 was higher than in other age groups. One report also pointed out that EPTB was particularly prevalent at younger ages (<25 years) (28). There was broad agreement that TP was primarily an immune reaction to tuberculin proteins in the pleural space (29). Unsurprisingly, the fact that older populations were not prone to TP may be attributed to the change in immune function in older adults (30). A basic survey of the global disease burden revealed that TB cases were predominant among youth, yet deaths were largely concentrated in older men (31). In addition, we found that the number of TP cases has risen rapidly among men 60 years or older in recent years. This suggests that TP control programs should mainly target young people and older men.

Our results indicated that the number of patients with TP was larger in eastern China, and most were farmers. Similarly, farmers also formed the bulk of China's PTB population (23, 32). Farmers constituted a high-risk subpopulation mainly due to tobacco use, educational levels, migration, and living environment (32). Future emphasis should be placed on reaching farmers in communities traditionally under-served by health services. In contrast to our finding, Chen et al. (33) found that TB highly prevailed in western China. A study on travel-related infections in mainland China showed that TB was common among Chinese laborers working abroad (34).

In general, the incidence rate and cases of TP have increased in mainland China from 2005 to 2018, with an average incidence of 2.5 per 100,000 population. The findings from our study are similar to the one conducted in Beijing, which also reported an increasing trend in TP that could be driven by direct transmission (35). The trend in China differs from that observed in other parts of the world. For instance, TP cases dropped in the US between 1993 and 2003. Similarly, in Barcelona, Spain, there was a drop in the number of cases from 2007 to 2014 (36, 37). Likewise, the incidence rate of tuberculous pleural effusions in Galicia, Spain, decreased from 9.6 per 100,000 population in 2000 to 4.8 per 100,000 population in 2009 (38). The annual hospitalization rate for TP in the US declined from 0.2 per 100,000 adult population in 2007 to 0.09 per 100,000 adult population in 2016 (39). Likely explanations for these reductions were medical factors, income, education, geography, environment, and habits (40). Overall, our data corresponds with the proportion of EPTB, which has spiked significantly in recent years in many countries. In China, the ratio of EPTB soared to 31.4, 10.3, and 20.0% in Beijing, Tianjin, and Shandong, respectively (36). Similar results have been reported in the EU and the European Economic Area (37). Until recently, the diagnosis of EPTB has been more challenging than PTB (39). Pleural fluid T-SPOT or interferon-gamma are currently being used to diagnose TP, and their value was equal (16). Therefore, improvement in economic conditions and advancements in the medical field has resulted in more persons with TP seeking medical help, resulting in an elevated diagnostic rate. Simultaneously, the prevalence of TB and PTB has declined over the past decade, owing to successful prevention and control measures (32).

The incidence of TP rose rapidly from 2005 to 2007, after which it grew steadily from 2007 to 2018. Several relative public health measures could partly explain this change. In 1993, the World Health Organization (WHO) declared TB a global emergency (40). Following this, China implemented the directly observed treatment (DOT), a short-course strategy based on international recommendations to address the TB problem in the 1990s, and covered the whole population after 2000 (41). The Law of Preventing and Controlling Infectious Disease was modified in 2004 (42), and an electronic Tuberculosis Information Management System (TBIMS) was established in 2005 (18). The TBIMS has played an essential role in increasing the reporting of TP cases since 2005. Before the national remit of the TBIMS, some cases in underdeveloped areas may have gone unregistered. Therefore, the actual APC from 2005 to 2007 may be lower in some areas due to the absence of the system in these areas. In addition, China launched major special national science and technology projects to prevent and control infectious diseases at the end of 2008 (43). Since then, TP incidence has proceeded at a slower and steady pace under effective management measures.

The annual spike in TP in China nearly coincided with spring. The spring peak was similar to the seasonality of PTB in Yunnan province (44) and Wuhan city (45). The TP seasonality may be due to the following factors: firstly, decreased vitamin D levels due to the lack of exposure to sunshine in winter, impaired host defense to TB (46), and boosted reactivation of latent TB (45). Considering that TB has a long incubation period varying from months to years (46), it is possible that infections occurred in the winter and manifested during spring, resulting in a peak in notification in spring. Second, the cold weather in winter delayed patients from seeking medical care. The effect of the Spring Festival holidays (usually in February or January) should also be considered, given that TP reporting sharply reduced during the Spring Festival month.

Tibet (11.4 per 100,000 population), Beijing (5.6 per 100,000 population), Xinjiang (5.1 per 100,000 population), and Inner Mongolia (4.9 per 100,000 population) had a higher mean annual incidence of TP. Likewise, the incidence of PTB among children was the highest in Tibet, with 15.95 per 100,000 children between 2009 and 2015 (46). Xinjiang, with the highest average annual incidence of PTB (135.03 per 100,000 population), was the most probable spatial and temporal cluster of TB (32, 47). The main reasons for the high TP burden in Tibet, Xinjiang, and Inner Mongolia included undeveloped economics, a high proportion of minorities, poor medical standards (48), and more agricultural population. Recent data from Xinjiang showed that the proportion of minorities positively impacted PTB incidence, but per capita GDP had a negative impact (49). Compared to the east and central regions in China, the west has a lower population density. Although a low population density aided in blocking the transmission of infectious diseases, it also increased the difficulty of healthcare professionals reaching people in need. Though Beijing was comparatively a thriving and prosperous city, the presence of immigrants played a much more significant role in the population. We speculated that the presence of immigrants was the principal factor explaining the high TP incidence in Beijing. Furthermore, our findings suggest that the current TP control strategy should focus on western or northern China.

Our research established an association between TP incidence, per capita medical expenses, and per capita GDP. After the patients were suspected or diagnosed with TP, they had to ask medical services for help, thus leading to costs in treatment. Posteriorly, a province with a high level of per capita GDP possessed better health care, superior housing conditions, and a good welfare system, ensuring the prompt diagnosis and treatment of TP. Zhang et al. studied TB incidence in seventeen cities of Shandong province and found that per capita medical expenditure affected reported TB incidence negatively (50). The difference in scope might explain this diversity between the two studies. Based on the current evidence, we did not find a significant relationship between TP incidence and population density, which disagreed with a previous study (51). Since PTB is contagious, especially in patients with sputum smear-positive on microscopy for acid–alcohol fast bacilli, it is possible that the discrepancy is likely because EPTB is usually not infectious (47).

Most TP is unilateral, and the right side is more susceptible. Pleural effusion is a common manifestation (52). TP accounts for <1% in western countries, and 30–80% of exudate occurs in developing countries such as India (53). Clinically, intravenous antibiotic administration, chest tube drainage, intrapleural administration of a fibrinolytic agent to dissolve fibrous adhesions, thoracotomy to remove fibrinous and infected tissue, and steroid therapy are remedies for pleural space infections (54). General guidelines recommend 6 months for EPTB treatment. Yet, a recent study proposed that the average duration of treatment for TP should be 8 months (36). A new study has added that smoking adversely influences treatment response and outcome in TB (55). Significantly, multidrug-resistant tuberculosis (MDR-TB) has been reported as a main issue in China compared to India and Indonesia (23). Pang et al. (35) observed substantial growth in MDR-TB, from 17.3 to 35.7%, for TP cases in China. Previous reports found high levels of mortality among people infected with MDR-TB, and improving greener space and air quality was said to attenuate the risk of death from TB/MDR- TB (56). Strengthening surveillance and ensuring follow-up at the clinic to ensure the treatment has been accomplished is very important to prevent the emergence of drug-resistant strains. A previous study contended that treatment completion rates of patients with EPTB were 90.9, 56.9, and 82.5% in Denmark, Finland, and Texas (US), respectively (37). The treatment success rate for EPTB was 70.1% in Accra, Ghana (57), and for TP was 90% in Pakistan (5). Furthermore, diagnosis and treatment should consider characteristics of the distribution of mycobacterium TB lineages in China. For example, the dominant strain in the east and central regions belonged to Lineage 2 (33). Bacillus Calmette-Guérin (BCG) was reported to protect against mycobacterium TB infection and advancement from infection to disease (58). BCG vaccination at birth which can halt TB suffering, represents a quantum leap in healthcare in China.

Based on the novel and original data, we uniquely analyzed the epidemiology of TP in China between 2005 and 2018, covering almost the entire population. However, this study was subject to some limitations. First, we only collected reported TP cases in mainland China, excluding PTB. Nevertheless, TP usually occurs secondary to PTB or is complicated with PTB, and a patient with both pulmonary and extrapulmonary TB should be classified as a case of PTB, causing incomplete data collection and analysis. Second, the diagnosis of TP might be underreported and may result in bias in TP registration, particularly in regions with restricted medical resources or ethnic minority groups, leading to an underestimation of the disease burden. Third, the retrospective study design has its inherent shortcoming in that no causal relationship can be inferred. Hence, the results of this study should be interpreted with caution. Future studies are required to validate our results, discover other contributing factors, and establish quick, convenient, and acceptable diagnostic methods for TP.

## Conclusion

An analysis of the long-term trend of patients registered with TP between 2005 and 2018 in mainland China showed an overall escalating tendency. Men, young adults (aged 15–29), and farmers were at high risk of contracting TP. Tibet, Beijing, Xinjiang, and Inner Mongolia had the highest clustering zones, and spring had the highest incidence among other seasons. In summary, clinicians need to be aware of this trend in incidence and enhance awareness of TP, particularly among the population at risk. Our study serves to optimize resource allocation in high-risk areas and formulate appropriate measures to alleviate the disease burden.

## Data availability statement

The datasets presented in this study can be found in online repositories. The names of the repository/repositories and accession number(s) can be found in the article/Supplementary material.

## Ethics statement

Ethical review and approval was not required for the study on human participants in accordance with the local legislation and institutional requirements. Written informed consent for participation was not required for this study in accordance with the national legislation and the institutional requirements.

## Author contributions

ZZ: had full access to the data in the study and took responsibility for the integrity of the data and the accuracy of the data analysis. ZZ and SC: concept and design. SC, YW, YZ, CL, QW, and JF: acquisition, analysis, and interpretation of data. SC and YW: drafting of the manuscript. ZZ, CL, and YL: revision of the manuscript. ZZ and HC: statistical analysis and supervision. All authors had full access to all the data in the study and accepted responsibility to submit for publication. All authors contributed to the article and approved the submitted version.
