# Supplementary material for: The incidence of tuberculous pleurisy in mainland China from 2005 to 2018
Source: Front Public Health. 2023 Jun 15;11:1180818. doi: 10.3389/fpubh.2023.1180818 (PMC10311513; doi:10.3389/fpubh.2023.1180818)
Supplement: Supplementary file 1 [file Data_Sheet_1.docx]

Supplementary Material

The incidence of tuberculous pleurisy in mainland China from 2005 to 2018

**Shuhan Chen, Yi Wang, Yuan Zhan, Changyu Liu, Qi Wang, Jie Feng, Huilong Chen, Zhilin Zeng**

**Correspondence:** Zhilin Zeng, ulfnssul@sina.com.

# Supplementary Table

Supplementary table 1. The incidence of TP in mainland China from 2005 to 2018

| Year | Incident cases | Incidece | National population size | Average incidence 2005-2018 | Average incidence 2005-2007 | Average incidence 2008-2018 |
| --- | --- | --- | --- | --- | --- | --- |
| 2005 | 5725 | 0.44 | 1299652859 | 2.51 | 1.64 | 2.80 |
| 2006 | 21494 | 1.64 | 1307559910 |  |  |  |
| 2007 | 28718 | 2.18 | 1314476400 |  |  |  |
| 2008 | 33365 | 2.53 | 1321290000 |  |  |  |
| 2009 | 39031 | 2.94 | 1328019999 |  |  |  |
| 2010 | 34561 | 2.59 | 1334740003 |  |  |  |
| 2011 | 33494 | 2.50 | 1340909996 |  |  |  |
| 2012 | 35270 | 2.62 | 1347349996 |  |  |  |
| 2013 | 35491 | 2.62 | 1354040000 |  |  |  |
| 2014 | 38124 | 2.81 | 1355133055 |  |  |  |
| 2015 | 39149 | 2.87 | 1362466686 |  |  |  |
| 2016 | 40076 | 2.92 | 1370784400 |  |  |  |
| 2017 | 42711 | 3.10 | 1379837956 |  |  |  |
| 2018 | 45711 | 3.29 | 1389096953 |  |  |  |

Note: TP, tuberculous pleurisy; incidence was calculated per 100,000 population.

Supplementary table 2. The incidence of TP in different province of mainland China from 2005 to 2018

| Name of province | Year | Incident cases | Incidence (per 100,000 population) |
| --- | --- | --- | --- |
| Bei Jing | 2005 | 210 | 1.37 |
|  | 2006 | 675 | 4.39 |
|  | 2007 | 915 | 5.79 |
|  | 2008 | 1103 | 6.75 |
|  | 2009 | 931 | 5.49 |
|  | 2010 | 1334 | 7.60 |
|  | 2011 | 1367 | 6.97 |
|  | 2012 | 1552 | 7.69 |
|  | 2013 | 1431 | 6.92 |
|  | 2014 | 1363 | 6.45 |
|  | 2015 | 1218 | 5.66 |
|  | 2016 | 1115 | 5.14 |
|  | 2017 | 974 | 4.48 |
|  | 2018 | 825 | 3.80 |
| Tian Jin | 2005 | 2 | 0.02 |
|  | 2006 | 10 | 0.10 |
|  | 2007 | 25 | 0.23 |
|  | 2008 | 12 | 0.11 |
|  | 2009 | 234 | 1.99 |
|  | 2010 | 317 | 2.58 |
|  | 2011 | 401 | 3.10 |
|  | 2012 | 377 | 2.78 |
|  | 2013 | 386 | 2.73 |
|  | 2014 | 319 | 2.17 |
|  | 2015 | 381 | 2.51 |
|  | 2016 | 330 | 2.13 |
|  | 2017 | 388 | 2.48 |
|  | 2018 | 376 | 2.42 |
|  | 2005 | 153 | 0.22 |
|  | 2006 | 710 | 1.04 |
|  | 2007 | 831 | 1.20 |
|  | 2008 | 969 | 1.40 |
|  | 2009 | 1076 | 1.54 |
|  | 2010 | 1083 | 1.54 |
|  | 2011 | 1507 | 2.10 |
|  | 2012 | 1983 | 2.74 |
|  | 2013 | 2167 | 2.97 |
|  | 2014 | 2612 | 3.56 |
|  | 2015 | 2779 | 3.76 |
|  | 2016 | 2901 | 3.91 |
|  | 2017 | 2983 | 3.99 |
|  | 2018 | 2707 | 3.60 |
| Shan xi | 2005 | 428 | 1.28 |
|  | 2006 | 1269 | 3.78 |
|  | 2007 | 1755 | 5.20 |
|  | 2008 | 2129 | 6.27 |
|  | 2009 | 1890 | 5.54 |
|  | 2010 | 1765 | 5.15 |
|  | 2011 | 1358 | 3.80 |
|  | 2012 | 1362 | 3.79 |
|  | 2013 | 1519 | 4.21 |
|  | 2014 | 1780 | 4.90 |
|  | 2015 | 1843 | 5.05 |
|  | 2016 | 1810 | 4.94 |
|  | 2017 | 1795 | 4.88 |
|  | 2018 | 1737 | 4.69 |
| Inner Mongolia | 2005 | 149 | 0.63 |
|  | 2006 | 596 | 2.50 |
|  | 2007 | 983 | 4.10 |
|  | 2008 | 1160 | 4.82 |
|  | 2009 | 2158 | 8.94 |
|  | 2010 | 1739 | 7.18 |
|  | 2011 | 1528 | 6.18 |
|  | 2012 | 1362 | 5.49 |
|  | 2013 | 1332 | 5.35 |
|  | 2014 | 1003 | 4.02 |
|  | 2015 | 1190 | 4.75 |
|  | 2016 | 1122 | 4.47 |
|  | 2017 | 1263 | 5.01 |
|  | 2018 | 1253 | 4.96 |
| Liao Ning | 2005 | 72 | 0.17 |
|  | 2006 | 307 | 0.73 |
|  | 2007 | 372 | 0.87 |
|  | 2008 | 438 | 1.02 |
|  | 2009 | 1614 | 3.74 |
|  | 2010 | 1809 | 4.19 |
|  | 2011 | 1983 | 4.53 |
|  | 2012 | 2551 | 5.82 |
|  | 2013 | 2140 | 4.88 |
|  | 2014 | 1942 | 4.42 |
|  | 2015 | 2121 | 4.83 |
|  | 2016 | 1679 | 3.83 |
|  | 2017 | 1553 | 3.55 |
|  | 2018 | 1719 | 3.93 |
| Ji Lin | 2005 | 66 | 0.24 |
|  | 2006 | 365 | 1.34 |
|  | 2007 | 479 | 1.76 |
|  | 2008 | 531 | 1.95 |
|  | 2009 | 1072 | 3.92 |
|  | 2010 | 902 | 3.29 |
|  | 2011 | 743 | 2.71 |
|  | 2012 | 566 | 2.06 |
|  | 2013 | 515 | 1.87 |
|  | 2014 | 545 | 1.98 |
|  | 2015 | 651 | 2.37 |
|  | 2016 | 524 | 1.90 |
|  | 2017 | 702 | 2.57 |
|  | 2018 | 687 | 2.53 |
| Hei Long Jiang | 2005 | 204 | 0.54 |
|  | 2006 | 925 | 2.42 |
|  | 2007 | 908 | 2.38 |
|  | 2008 | 994 | 2.60 |
|  | 2009 | 855 | 2.24 |
|  | 2010 | 723 | 1.89 |
|  | 2011 | 657 | 1.71 |
|  | 2012 | 577 | 1.50 |
|  | 2013 | 531 | 1.38 |
|  | 2014 | 421 | 1.10 |
|  | 2015 | 397 | 1.04 |
|  | 2016 | 361 | 0.95 |
|  | 2017 | 585 | 1.54 |
|  | 2018 | 615 | 1.62 |
| Shang Hai | 2005 | 38 | 0.21 |
|  | 2006 | 478 | 2.69 |
|  | 2007 | 499 | 2.75 |
|  | 2008 | 390 | 2.10 |
|  | 2009 | 776 | 4.11 |
|  | 2010 | 723 | 3.76 |
|  | 2011 | 699 | 3.04 |
|  | 2012 | 700 | 2.98 |
|  | 2013 | 670 | 2.81 |
|  | 2014 | 592 | 2.45 |
|  | 2015 | 505 | 2.08 |
|  | 2016 | 518 | 2.14 |
|  | 2017 | 452 | 1.87 |
|  | 2018 | 429 | 1.77 |
| Jiang Su | 2005 | 232 | 0.31 |
|  | 2006 | 910 | 1.22 |
|  | 2007 | 1230 | 1.63 |
|  | 2008 | 1432 | 1.88 |
|  | 2009 | 1433 | 1.87 |
|  | 2010 | 1412 | 1.83 |
|  | 2011 | 1507 | 1.92 |
|  | 2012 | 1849 | 2.34 |
|  | 2013 | 1749 | 2.21 |
|  | 2014 | 2165 | 2.73 |
|  | 2015 | 2124 | 2.67 |
|  | 2016 | 2183 | 2.74 |
|  | 2017 | 2122 | 2.65 |
|  | 2018 | 2036 | 2.54 |
| Zhe Jiang | 2005 | 291 | 0.60 |
|  | 2006 | 897 | 1.83 |
|  | 2007 | 1117 | 2.24 |
|  | 2008 | 1381 | 2.73 |
|  | 2009 | 2799 | 5.47 |
|  | 2010 | 2645 | 5.11 |
|  | 2011 | 2809 | 5.16 |
|  | 2012 | 2932 | 5.37 |
|  | 2013 | 2833 | 5.17 |
|  | 2014 | 2672 | 4.86 |
|  | 2015 | 2349 | 4.26 |
|  | 2016 | 2301 | 4.15 |
|  | 2017 | 2587 | 4.63 |
|  | 2018 | 2472 | 4.37 |
| An Hui | 2005 | 91 | 0.15 |
|  | 2006 | 400 | 0.65 |
|  | 2007 | 549 | 0.90 |
|  | 2008 | 607 | 0.99 |
|  | 2009 | 707 | 1.15 |
|  | 2010 | 807 | 1.32 |
|  | 2011 | 927 | 1.56 |
|  | 2012 | 902 | 1.51 |
|  | 2013 | 1102 | 1.84 |
|  | 2014 | 1398 | 2.32 |
|  | 2015 | 1370 | 2.25 |
|  | 2016 | 1435 | 2.34 |
|  | 2017 | 1716 | 2.77 |
|  | 2018 | 1944 | 3.11 |
| Fu Jian | 2005 | 104 | 0.30 |
|  | 2006 | 267 | 0.76 |
|  | 2007 | 356 | 1.00 |
|  | 2008 | 427 | 1.19 |
|  | 2009 | 528 | 1.45 |
|  | 2010 | 499 | 1.38 |
|  | 2011 | 489 | 1.33 |
|  | 2012 | 377 | 1.01 |
|  | 2013 | 416 | 1.11 |
|  | 2014 | 383 | 1.01 |
|  | 2015 | 398 | 1.05 |
|  | 2016 | 442 | 1.15 |
|  | 2017 | 506 | 1.31 |
|  | 2018 | 664 | 1.70 |
| Jiang Xi | 2005 | 237 | 0.55 |
|  | 2006 | 895 | 2.08 |
|  | 2007 | 991 | 2.28 |
|  | 2008 | 1106 | 2.53 |
|  | 2009 | 1618 | 3.68 |
|  | 2010 | 1469 | 3.31 |
|  | 2011 | 1154 | 2.59 |
|  | 2012 | 1207 | 2.69 |
|  | 2013 | 1143 | 2.54 |
|  | 2014 | 1171 | 2.59 |
|  | 2015 | 1195 | 2.63 |
|  | 2016 | 1564 | 3.43 |
|  | 2017 | 1571 | 3.42 |
|  | 2018 | 1598 | 3.46 |
| Shan Dong | 2005 | 222 | 0.24 |
|  | 2006 | 1235 | 1.34 |
|  | 2007 | 1748 | 1.88 |
|  | 2008 | 1805 | 1.93 |
|  | 2009 | 1714 | 1.82 |
|  | 2010 | 1609 | 1.70 |
|  | 2011 | 1170 | 1.22 |
|  | 2012 | 1348 | 1.40 |
|  | 2013 | 1324 | 1.37 |
|  | 2014 | 1492 | 1.53 |
|  | 2015 | 1509 | 1.54 |
|  | 2016 | 1583 | 1.61 |
|  | 2017 | 1821 | 1.83 |
|  | 2018 | 2045 | 2.04 |
| He Nan | 2005 | 273 | 0.29 |
|  | 2006 | 683 | 0.73 |
|  | 2007 | 1215 | 1.29 |
|  | 2008 | 1155 | 1.23 |
|  | 2009 | 4111 | 4.36 |
|  | 2010 | 3856 | 4.06 |
|  | 2011 | 3864 | 4.11 |
|  | 2012 | 4131 | 4.40 |
|  | 2013 | 4396 | 4.67 |
|  | 2014 | 4929 | 5.24 |
|  | 2015 | 4997 | 5.30 |
|  | 2016 | 4836 | 5.10 |
|  | 2017 | 4517 | 4.74 |
|  | 2018 | 4105 | 4.29 |
| Hu Bei | 2005 | 198 | 0.35 |
|  | 2006 | 742 | 1.30 |
|  | 2007 | 821 | 1.44 |
|  | 2008 | 745 | 1.31 |
|  | 2009 | 654 | 1.15 |
|  | 2010 | 596 | 1.04 |
|  | 2011 | 653 | 1.14 |
|  | 2012 | 521 | 0.90 |
|  | 2013 | 446 | 0.77 |
|  | 2014 | 466 | 0.80 |
|  | 2015 | 489 | 0.84 |
|  | 2016 | 548 | 0.94 |
|  | 2017 | 860 | 1.46 |
|  | 2018 | 1247 | 2.11 |
| Hu Nan | 2005 | 199 | 0.32 |
|  | 2006 | 771 | 1.22 |
|  | 2007 | 1236 | 1.95 |
|  | 2008 | 1454 | 2.29 |
|  | 2009 | 1055 | 1.65 |
|  | 2010 | 1068 | 1.67 |
|  | 2011 | 844 | 1.28 |
|  | 2012 | 932 | 1.41 |
|  | 2013 | 848 | 1.28 |
|  | 2014 | 868 | 1.30 |
|  | 2015 | 799 | 1.19 |
|  | 2016 | 774 | 1.14 |
|  | 2017 | 1074 | 1.57 |
|  | 2018 | 1563 | 2.28 |
| Guang Dong | 2005 | 254 | 0.28 |
|  | 2006 | 862 | 0.94 |
|  | 2007 | 1215 | 1.31 |
|  | 2008 | 1531 | 1.62 |
|  | 2009 | 1472 | 1.54 |
|  | 2010 | 1404 | 1.46 |
|  | 2011 | 1398 | 1.34 |
|  | 2012 | 1391 | 1.32 |
|  | 2013 | 1479 | 1.40 |
|  | 2014 | 1624 | 1.53 |
|  | 2015 | 1686 | 1.57 |
|  | 2016 | 2026 | 1.87 |
|  | 2017 | 2231 | 2.03 |
|  | 2018 | 2152 | 1.93 |
| Guang Xi | 2005 | 302 | 0.65 |
|  | 2006 | 874 | 1.88 |
|  | 2007 | 999 | 2.12 |
|  | 2008 | 1065 | 2.23 |
|  | 2009 | 1214 | 2.52 |
|  | 2010 | 884 | 1.82 |
|  | 2011 | 879 | 1.91 |
|  | 2012 | 1066 | 2.29 |
|  | 2013 | 1011 | 2.16 |
|  | 2014 | 943 | 2.00 |
|  | 2015 | 851 | 1.79 |
|  | 2016 | 1161 | 2.42 |
|  | 2017 | 1324 | 2.74 |
|  | 2018 | 1328 | 2.72 |
| Hai Nan | 2005 | 15 | 0.18 |
|  | 2006 | 45 | 0.54 |
|  | 2007 | 62 | 0.74 |
|  | 2008 | 40 | 0.47 |
|  | 2009 | 42 | 0.49 |
|  | 2010 | 26 | 0.30 |
|  | 2011 | 29 | 0.33 |
|  | 2012 | 34 | 0.39 |
|  | 2013 | 26 | 0.29 |
|  | 2014 | 45 | 0.50 |
|  | 2015 | 39 | 0.43 |
|  | 2016 | 61 | 0.67 |
|  | 2017 | 81 | 0.88 |
|  | 2018 | 241 | 2.40 |
| Chong Qing | 2005 | 174 | 0.62 |
|  | 2006 | 608 | 2.17 |
|  | 2007 | 623 | 2.22 |
|  | 2008 | 921 | 3.27 |
|  | 2009 | 1047 | 3.69 |
|  | 2010 | 875 | 3.06 |
|  | 2011 | 843 | 2.92 |
|  | 2012 | 926 | 3.17 |
|  | 2013 | 740 | 2.51 |
|  | 2014 | 856 | 2.88 |
|  | 2015 | 916 | 3.06 |
|  | 2016 | 979 | 3.25 |
|  | 2017 | 1235 | 4.05 |
|  | 2018 | 1534 | 4.99 |
| Si Chuan | 2005 | 653 | 0.80 |
|  | 2006 | 2253 | 2.74 |
|  | 2007 | 2827 | 3.46 |
|  | 2008 | 3328 | 4.09 |
|  | 2009 | 2114 | 2.58 |
|  | 2010 | 1780 | 2.17 |
|  | 2011 | 1847 | 2.30 |
|  | 2012 | 1745 | 2.17 |
|  | 2013 | 1934 | 2.39 |
|  | 2014 | 2106 | 2.60 |
|  | 2015 | 2490 | 3.06 |
|  | 2016 | 2555 | 3.11 |
|  | 2017 | 2884 | 3.49 |
|  | 2018 | 3414 | 4.11 |
| Gui Zhou | 2005 | 375 | 1.01 |
|  | 2006 | 1240 | 3.32 |
|  | 2007 | 2045 | 5.44 |
|  | 2008 | 2670 | 7.10 |
|  | 2009 | 2319 | 6.12 |
|  | 2010 | 1091 | 2.87 |
|  | 2011 | 1005 | 2.89 |
|  | 2012 | 1066 | 3.07 |
|  | 2013 | 1129 | 3.24 |
|  | 2014 | 1388 | 3.96 |
|  | 2015 | 1445 | 4.12 |
|  | 2016 | 1455 | 4.12 |
|  | 2017 | 1451 | 4.08 |
|  | 2018 | 1868 | 5.22 |
| Yun Nan | 2005 | 130 | 0.29 |
|  | 2006 | 413 | 0.93 |
|  | 2007 | 871 | 1.94 |
|  | 2008 | 1025 | 2.27 |
|  | 2009 | 913 | 2.01 |
|  | 2010 | 601 | 1.31 |
|  | 2011 | 751 | 1.63 |
|  | 2012 | 732 | 1.58 |
|  | 2013 | 846 | 1.82 |
|  | 2014 | 885 | 1.89 |
|  | 2015 | 1127 | 2.39 |
|  | 2016 | 1219 | 2.57 |
|  | 2017 | 1200 | 2.52 |
|  | 2018 | 1508 | 3.14 |
| Tibet | 2005 | 4 | 0.15 |
|  | 2006 | 10 | 0.36 |
|  | 2007 | 4 | 0.14 |
|  | 2008 | 3 | 0.11 |
|  | 2009 | 380 | 13.24 |
|  | 2010 | 359 | 12.38 |
|  | 2011 | 342 | 11.40 |
|  | 2012 | 419 | 13.81 |
|  | 2013 | 441 | 14.32 |
|  | 2014 | 545 | 17.47 |
|  | 2015 | 536 | 16.88 |
|  | 2016 | 607 | 18.74 |
|  | 2017 | 585 | 17.67 |
|  | 2018 | 795 | 23.58 |
| Shaan Xi | 2005 | 231 | 0.62 |
|  | 2006 | 1132 | 3.04 |
|  | 2007 | 1559 | 4.17 |
|  | 2008 | 1718 | 4.58 |
|  | 2009 | 1864 | 4.95 |
|  | 2010 | 1726 | 4.58 |
|  | 2011 | 1470 | 3.94 |
|  | 2012 | 1450 | 3.87 |
|  | 2013 | 1689 | 4.50 |
|  | 2014 | 2011 | 5.34 |
|  | 2015 | 1997 | 5.29 |
|  | 2016 | 2209 | 5.82 |
|  | 2017 | 2158 | 5.66 |
|  | 2018 | 1914 | 4.99 |
| Gan Su | 2005 | 138 | 0.54 |
|  | 2006 | 519 | 2.00 |
|  | 2007 | 706 | 2.71 |
|  | 2008 | 950 | 3.63 |
|  | 2009 | 446 | 1.70 |
|  | 2010 | 205 | 0.78 |
|  | 2011 | 132 | 0.52 |
|  | 2012 | 112 | 0.44 |
|  | 2013 | 139 | 0.54 |
|  | 2014 | 135 | 0.52 |
|  | 2015 | 246 | 0.95 |
|  | 2016 | 339 | 1.30 |
|  | 2017 | 588 | 2.25 |
|  | 2018 | 763 | 2.91 |
| Qing Hai | 2005 | 5 | 0.09 |
|  | 2006 | 28 | 0.52 |
|  | 2007 | 37 | 0.68 |
|  | 2008 | 92 | 1.67 |
|  | 2009 | 408 | 7.36 |
|  | 2010 | 324 | 5.81 |
|  | 2011 | 272 | 4.83 |
|  | 2012 | 252 | 4.44 |
|  | 2013 | 216 | 3.77 |
|  | 2014 | 278 | 4.81 |
|  | 2015 | 273 | 4.68 |
|  | 2016 | 314 | 5.34 |
|  | 2017 | 355 | 5.99 |
|  | 2018 | 597 | 9.98 |
| Ning Xia | 2005 | 10 | 0.17 |
|  | 2006 | 124 | 2.08 |
|  | 2007 | 137 | 2.27 |
|  | 2008 | 160 | 2.62 |
|  | 2009 | 90 | 1.49 |
|  | 2010 | 46 | 0.74 |
|  | 2011 | 79 | 1.25 |
|  | 2012 | 117 | 1.83 |
|  | 2013 | 77 | 1.19 |
|  | 2014 | 114 | 1.74 |
|  | 2015 | 96 | 1.45 |
|  | 2016 | 86 | 1.29 |
|  | 2017 | 81 | 1.20 |
|  | 2018 | 192 | 2.82 |
| Xin Jiang | 2005 | 266 | 1.34 |
|  | 2006 | 1251 | 6.22 |
|  | 2007 | 1603 | 7.82 |
|  | 2008 | 2024 | 9.66 |
|  | 2009 | 1497 | 7.03 |
|  | 2010 | 884 | 4.10 |
|  | 2011 | 787 | 3.61 |
|  | 2012 | 731 | 3.31 |
|  | 2013 | 816 | 3.65 |
|  | 2014 | 1074 | 4.74 |
|  | 2015 | 1132 | 4.93 |
|  | 2016 | 1039 | 4.40 |
|  | 2017 | 1069 | 4.46 |
|  | 2018 | 1383 | 5.66 |

Note: TP, tuberculous pleurisy.
